# Evaluation of Retinal Pigment Epithelial Cell Cytotoxicity of Recombinant Tissue Plasminogen Activator Using Human-Induced Pluripotent Stem Cells

**DOI:** 10.1155/2019/7189241

**Published:** 2019-03-19

**Authors:** Hiroyuki Kamao, Atsushi Miki, Junichi Kiryu

**Affiliations:** Department of Ophthalmology, Kawasaki Medical School, 577 Matsushima, Kurashiki, Okayama 701-0114, Japan

## Abstract

**Purpose:**

The evaluation of drug-induced cytotoxicity is of great importance for the clinical application of pharmaceutical products, and human-induced pluripotent stem cells (hiPSCs) have received considerable scrutiny as a cell source for in vitro cytotoxicity testing. The aim of this study is to validate the concept of cytotoxicity testing using hiPSC-derived retinal pigment epithelium (hiPSC-RPE) by comparing the responsiveness of human fetal RPE (hfRPE) and human RPE cell line (ARPE19) to recombinant tissue plasminogen activator (rtPA).

**Methods:**

HfRPE, two types of hiPSC-RPE, and ARPE19 were cultured in media with or without rtPA. A lactate dehydrogenase release assay was performed to investigate the dose- and time-dependent effects of rtPA on cell death. RPE function was evaluated by measuring the secretion of pigment epithelium-derived factor (PEDF) and vascular endothelial growth factor (VEGF) and RPE-specific gene expression.

**Results:**

Rates of cell damage in hfRPE and both hiPS-RPE were increased by rtPA supplementation (2000 and 4000 *μ*g/ml) for 1 hour, whereas ARPE19 cell damage was increased by supplementation with rtPA at concentrations higher than 50 *μ*g/ml. Although 100 *μ*g/ml rtPA for 24 hours did not affect RPE cell function, sustained rtPA exposure induced prolonged cytotoxic effects in hfRPE and two hiPSC-RPE, but not ARPE19.

**Conclusion:**

The responsiveness of hiPSC-RPE to rtPA is similar to that of hfRPE in terms of cell death and cell function. Thus, hiPSC-RPE is a valuable cell source for in vitro cytotoxicity testing.

## 1. Introduction

The evaluation of drug-induced cytotoxicity is of great importance for the clinical application of pharmaceutical products. Although human cell lines or primary rodent cells have been used for cytotoxicity testing in vitro, these cells might possess different characteristics from native human cells due to immortalization or different race. In recent years, human-induced pluripotent stem cells (hiPSCs) [1] have received considerable attention as a potential cell source for pharmaceutical development. HiPSCs were generated from somatic cells by reprogramming and generate virtually any cell type in the human body in culture; thus, hiPSCs provide a more efficient assay system for the evaluation of drug-induced cytotoxicity and efficacy. We previously established hiPSC-derived retinal pigment epithelium (hiPSC-RPE) [2, 3] and transplanted an autologous hiPSC-RPE cell sheet into a patient with age-related macular degeneration [4]. Although few published reports have investigated the availability of hiPSC-RPE for drug-induced cytotoxicity testing, these cells have the potential to improve the accuracy of RPE toxicity analysis.

Subretinal hemorrhage is the presence of a blood clot between the photoreceptors and RPE and typically results in poor visual outcomes [5]. To address this health issue, various interventions to remove blood clots have been tested, including the recent use of recombinant tissue plasminogen activator (rtPA) [6, 7]. The rtPA is a thrombolytic agent and is used to treat various forms of thrombosis such as ischaemic stroke, myocardial infarction, and pulmonary embolism. Specifically, rtPA dissolves blood clots via fibrin lysis, and isolating hemorrhage was displaced from the macula. However, rtPA demonstrates retinal toxicity in vivo: animal experiments revealed necrotic retinal holes and loss of photoreceptors in rabbits [8] and a case report described diffuse pigmentary alterations due to RPE toxicity [7, 9]. Based on these reports, rtPA at a dose greater than 100 *μ*g/ml appears to be toxic to the neural retina and RPE. In vitro, rtPA damages nerve cells in the ganglion cell layer or inner nuclear layer by enhancing *N*-methyl-D-aspartate signalling and apoptosis [10, 11]. However, studies investigating rtPA-induced RPE toxicity are limited.

In the present study, we compared the responsiveness of hiPSC-RPE, human fetal RPE (hfRPE) as native RPE, and a human RPE cell line (ARPE19) to rtPA by evaluating cell morphology, cell death, and cell function to validate the concept of cytotoxicity testing using hiPSC-RPE. In addition, the potential effects of rtPA administration on cell function were elucidated by evaluating rtPA cytotoxicity in vitro at clinically used and toxic concentrations.

## 2. Materials and Methods

### 2.1. Culture of hiPSCs, hiPSC-RPE, hfRPE, and ARPE19

The hiPS cell lines 454E2 [12] and 253G1 [13], which were derived from healthy human dental pulp cells using six transcription factors (Oct3/4, Sox2, Klf4, L-Myc, Lin28, and p53) and dermal fibroblast cells using three transcription factors (Oct3/4, Sox2, and Klf4), respectively, were supplied by RIKEN BioResource Center (Ibaraki, Japan). HfRPE and ARPE19 were purchased from Lonza (Basel, Switzerland) and the ATCC (Manassas, VA, USA), respectively. The methods used for hiPSC maintenance and differentiation have been previously described [14]. RPE were cultured on CELLstart-coated (GIbcO, Carlsbad, CA, USA) dishes in preconfluent medium (F10 (Sigma-Aldrich Corp., St. Louis, MO, USA) with 10% fetal bovine serum) before reaching confluence and in postconfluent medium (DMEM/F12 (7 : 3) supplemented with B27 (Invitrogen, Carlsbad, CA, USA), 2 mM L-glutamine (Sigma-Aldrich Corp.), 10 ng/mL basic fibroblast growth factor (Wako, Osaka, Japan), and SB431542 (0.5 *μ*M, Sigma-Aldrich Corp.)) after reaching confluence. The medium was changed every 2 to 3 days. Cultured RPE were recorded using a laser scanning confocal microscope (IX81; Olympus, Tokyo, Japan).

### 2.2. Cytotoxicity Assay

The cell damage rate was measured by performing a lactate dehydrogenase (LDH) cytotoxicity test (Wako) following the manufacturer's instructions. rtPA (monteplase) was purchased from Eisai (Tokyo, Japan). RPE in preconfluent medium were seeded at a density of 1.0 × 10^5^ cells/cm^2^ on 96-well plates, and preconfluent medium was switched to postconfluent medium for 2 weeks after confluence was reached. RPE were cultured in postconfluent medium with eight different dilutions of rtPA (0, 10, 20, 50, 100, 1000, 2000, and 4000 *μ*g/ml) for 1 hour or 24 hours, and culture supernatants were evaluated at 1, 7, and 28 days after rtPA administration (each *n*=5). The absorbance of each supernatant treated with a chemiluminescent reagent was recorded using a multimode microplate reader (Varioskan®; Thermo Scientific), and the cell damage rate was calculated with the following equation:

(1) $$\begin{array}{c} \text{cell damage rate}\left(\%\right)=\frac{\left[\left(\text{sample absorbance}\right)-\left(\text{negative control absorbance}\right)\right]}{\left[\left(\text{positive control absorbance}\right)-\left(\text{negative control absorbance}\right)\right]}\times 100. \end{array}$$

The positive control was RPE supernatant treated with 0.2% Tween 20 for 45 min at 37°C.

### 2.3. Scanning Electron Microscopy

RPE grown in Transwell® inserts were fixed in 2.5% glutaraldehyde, postfixed with 1% osmium tetroxide, dehydrated with increasing concentrations of ethanol, and visualized with a scanning electron microscope (S-3400N, Hitachi, Japan).

### 2.4. Immunofluorescence Assays

The methods used for RPE immunocytochemistry have been previously described [3]. ZO-1 was detected with a primary antibody (rabbit; Zymed Thermo Fisher Scientific, Waltham, MA, USA; 1/100). Bound primary antibodies were detected with Alexa Fluor 488-labeled goat anti-rabbit IgG secondary antibodies (Invitrogen, 1/500), and nuclei were stained with 4′,6-diamidino-2-phenylindole (DAPI) (1 *μ*g/ml; Molecular Probes, Thermo Fisher Scientific). Samples were imaged using a laser scanning confocal microscope (FV1000-D; Olympus, Tokyo, Japan).

### 2.5. Enzyme-Linked Immunosorbent Assay (ELISA) to Measure Pigment Epithelium-Derived Factor (PEDF) and Vascular Endothelial Growth Factor (VEGF) Secretion

The culture medium from confluent RPE treated with and without rtPA was collected 24 hours after the medium was changed (each *n*=3). PEDF [15] and VEGF [16] secretion levels were measured using a human PEDF-ELISA kit (BioVender, Modřice, Czech Republic) and a human VEGF-ELISA kit (eBioscience, San Diego, CA, USA), respectively, following the manufacturers' instructions.

### 2.6. Real-Time Quantitative Reverse Transcription Polymerase Chain Reaction (Real-Time qRT-PCR)

Total RNA was extracted from RPE treated with and without rtPA using an RNeasy Plus Mini Kit® (QIAGEN, Hilden, Germany) according to the manufacturer's instructions (each *n*=3). RNA concentration and quality were assessed with a NanoDrop 1000 spectrophotometer (Thermo Scientific). RNA was reverse transcribed in a 20 *μ*l reaction containing 11 *μ*l of RNA (1000 ng) in DNase- and RNase-free water (QIAGEN), 1 *μ*l of 50 *μ*M Oligo (dT) 20 (Invitrogen), 1 *μ*l of 10 mM dNTP Mix (Invitrogen), 1 *μ*l of RNaseOUT (20 U/*μ*l; Invitrogen), and SuperScript™ III Reverse Transcriptase (4 *μ*l of 5× First-Standard Buffer, 1 *μ*l of 0.1 M DTT, and 1 *μ*l of SuperScript III at 200 U/*μ*l; Invitrogen). cDNA synthesis was performed using the following reaction conditions: 5 min at 25°C, 60 min at 50°C, and 15 min at 70°C. Reverse transcription PCR reactions were performed using Ex Taq® DNA polymerase (Takara Bio, Shiga, Japan), and real-time PCR was performed using SYBRTM Green master mix (Applied Biosystems, Foster City, CA, USA). The following thermal cycling conditions were applied: one cycle at 50°C for 120 sec and 95°C for 120 sec, followed by 40 cycles at 95°C for 15 sec and 58°C for 60 sec. Relative gene expression of RPE65, VMD2, RLBP1, and MERTK was determined using the 2^−(ΔΔCt)^ method, with TFRC as a housekeeping gene which was unaffected by rtPA administration. The following forward and reverse primer (FP and RP) sequences (Fasmac, Kanagawa, Japan, and Takara Bio Inc., Siga, Japan) were used to amplify four RPE-specific genes (RPE65: FP; CATACCCATCAGAACCCATCTTTG, RP; CCTTGGCATTCAGAATCAGGAG, VMD2: FP; GCCAGGTGTTGGTCCTTTGTC, RP; GCGTCCACAGCCTTAAGCTTC, RLBP1: FP; GCCCTGACTTCCTATCCTAGGGAAG, RP; GAGAAGTAAGGAGGGAGGGAGAGGG, and MERTK: FP; AGGTTGAAGCAGCCCGAAGA, RP; TGCTTGGTTCCGAACGTCAG) and two housekeeping genes (TFRC: FP; GCGAGCACTGACCAGATAAGAATG, RP; TCCCGATAATGTGTTAGGATTGTGA and HPRT1: FP; GGCAGTATAATCCAAAGATGGTCAA, RP; GTCAAGGGCATATCCTACAACAAAC).

### 2.7. Statistical Analysis

Values are expressed as the mean ± SEM, and *P* < 0.05 was considered statistically significant (asterisks, *P* < 0.01). LDH release assay, PEDF and VEGF secretion, and relative RPE-specific gene expression were analyzed by performing one-way analysis of variance (ANOVA) followed by Scheffe's test.

## 3. Results

### 3.1. rtPA Cytotoxicity in RPE

**Figure 1:**
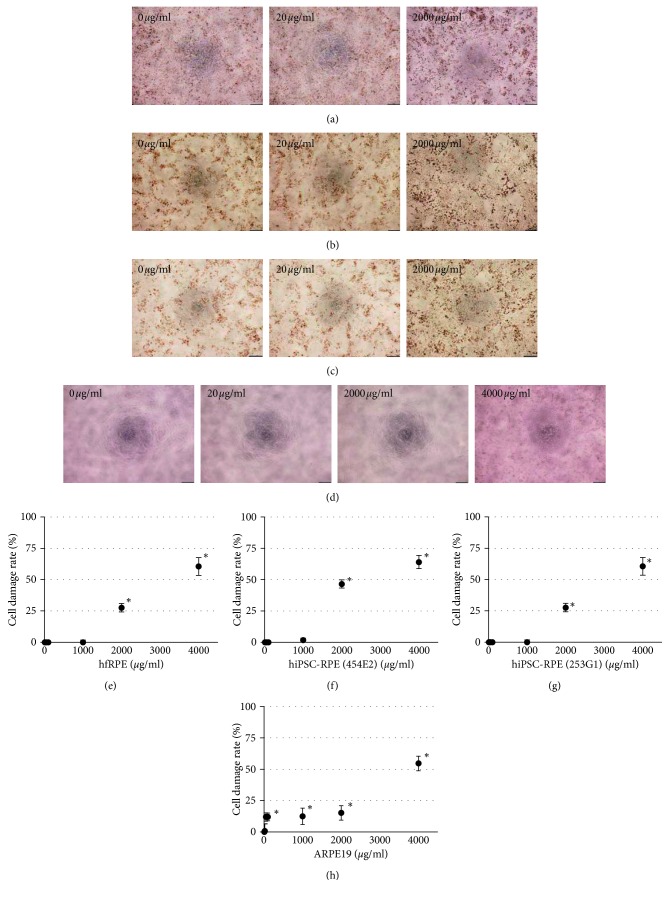
Cytotoxicity of 1-hour rtPA exposure in RPE. Phase-contrast images of hfRPE (a), hiPSC-RPE ((b) 454E2), hiPSC-RPE ((c) 253G1), and ARPE19 (d). Scale bar, 200 mm. Cell damage rate of eight different dilutions in hfRPE (e), hiPSC-RPE ((f) 454E2), hiPSC-RPE ((g) 253G1), and ARPE19 (h); *n*=5 for each; ^*∗*^*P* < 0.01.

A morphological and LDH release assay was performed to evaluate rtPA-induced cytotoxicity in hfRPE, two different hiPSC-RPE (253G1 and 454E2), and ARPE19. All RPE were treated with eight different dilutions of rtPA (0, 10, 20, 50, 100, 1000, 2000, and 4000 *μ*g/ml) for 1 hour, and each RPE and corresponding culture supernatants were evaluated 1 day after rtPA administration. HfRPE treated with 0 to 1000 *μ*g/ml rtPA showed no obvious morphological changes although floating cells were observed in the medium containing 2000 and 4000 *μ*g/ml rtPA (Figure 1(a)). Both hiPSC-RPE exhibited results comparable to hfRPE (Figures 1(b) and 1(c)), whereas ARPE19 treated with rtPA at 2000 *μ*g/ml or less showed no morphological changes, and floating cells were observed in the medium containing 4000 *μ*g/ml rtPA (Figure 1(d)). Based on the LDH release assay, the cell damage rates of hfRPE treated with 2000 and 4000 *μ*g/ml rtPA were significantly increased although there were no significant differences between rtPA-treated hfRPE and control at other concentrations (Figure 1(e)). We obtained similar results with both hiPSC-RPE, whereas the cell damage rates of ARPE19 treated with rtPA at concentrations greater than 50 *μ*g/ml were significantly increased (Figure 1(e)). Based on these in vitro cytotoxicity assay results, the responsiveness of hiPSC-RPE, but not ARPE19, to rtPA is similar to that of hfRPE.

**Figure 2:**
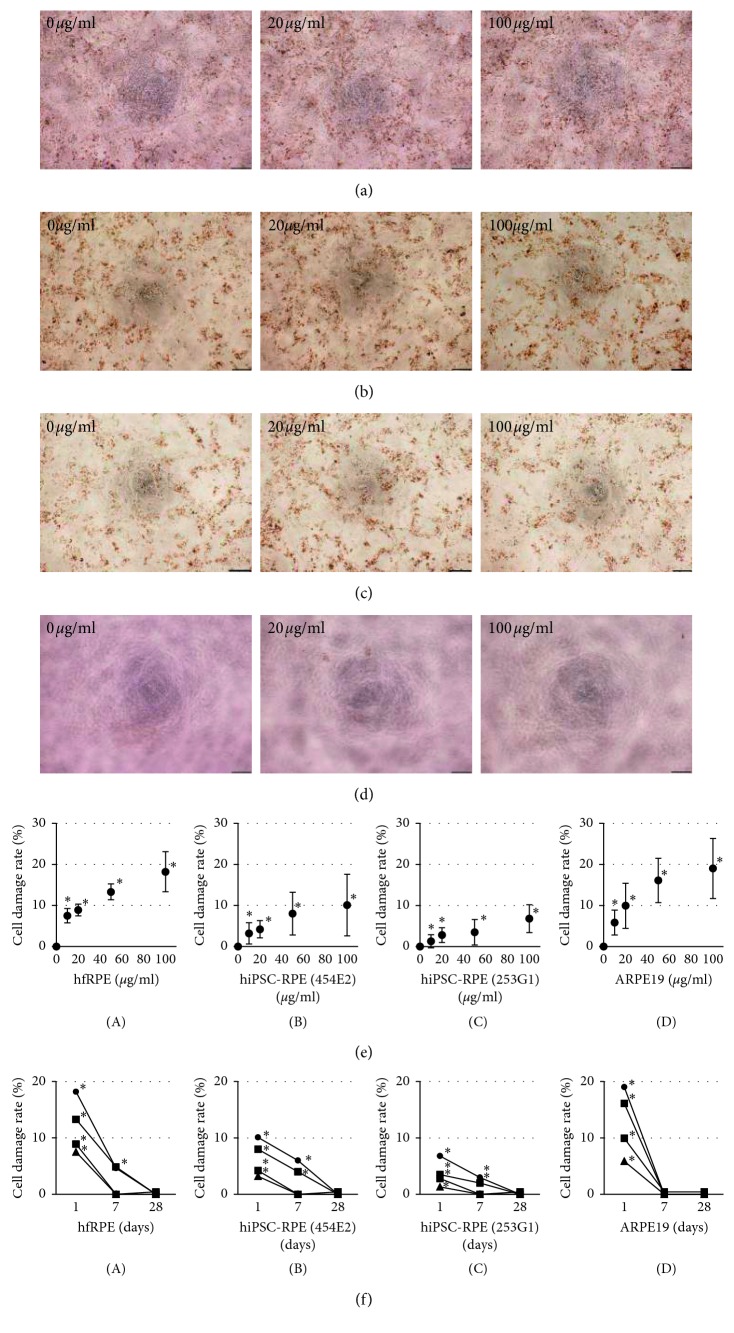
Cytotoxicity of 24-hour rtPA exposure in RPE. Phase-contrast images of hfRPE (a) hiPSC-RPE ((b) 454E2), hiPSC-RPE ((c) 253G1), and ARPE19 (d). Scale bar, 200 mm. (e) Cell damage rate of five different dilutions in hfRPE (A), hiPSC-RPE ((B) 454E2), hiPSC-RPE ((C) 253G1), and ARPE19 (D); *n*=5 for each; ^*∗*^*P* < 0.01. (f) Time course of the cell damage rate of five different dilutions in hfRPE (A), hiPSC-RPE ((B) 454E2), hiPSC-RPE ((C) 253G1), and ARPE19 (D); *n*=5 for each; ^*∗*^*P* < 0.01.

Next, we investigated whether sustained rtPA exposure affects cell death. Previous reports showed that rtPA at a dose greater than 100 *μ*g/ml appears to be toxic to RPE, and thus, all RPE were cultured with five different dilutions of rtPA (0, 10, 20, 50, and 100 *μ*g/ml) for 24 hours. Each culture condition and supernatant was evaluated at 1, 7, and 28 days after rtPA administration. All RPE exhibited no morphological changes at all time points examined (Figures 2(a)–2(d)). As demonstrated in the LDH release assay, the cell damage rates of all rtPA-treated hfRPE were significantly increased on day 1 (Figure 2(e)), and 50 and 100 *μ*g/ml rtPA induced cell damage on day 7 (Figure 2(f)). The two hiPSC-RPE showed results consistent with hfRPE (Figures 2(e) and 2(f)). The cell damage rates of all rtPA-treated ARPE19 were significantly increased on day 1; however, there were no significant differences between rtPA-treated ARPE19 cells and control on days 7 and 28 (Figures 2(e) and 2(f)). The clinically toxic concentration (100 *μ*g/ml) induced evidence of prolonged cytotoxic effects in hfRPE and the two hiPSC-RPE but not ARPE19.

### 3.2. Effects of 24-Hour rtPA Exposure on Cell Morphology and RPE-Specific Function

**Figure 3:**
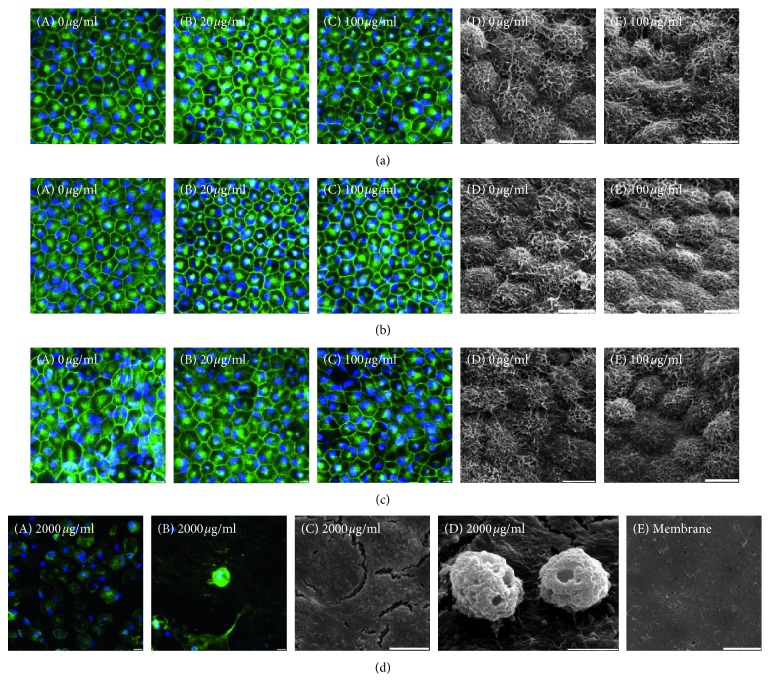
Effects of 24-hour rtPA exposure on cell morphology. ZO-1-stained (green) and DAPI-stained (blue) confocal images ((A) 0 *μ*g/ml, (B) 20 *μ*g/ml, and (C) 100 *μ*g/ml) and scanning electron microscopy images ((D) 0 *μ*g/ml and (E) 100 *μ*g/ml) of hfRPE (a), hiPSC-RPE ((b) 454E2), and hiPSC-RPE ((c) 253G1) treated with 0, 20, and 100 *μ*g/ml rtPA. Scale bar, 10 mm. (d) ZO-1-stained (green) and DAPI-stained (blue) stained confocal images ((A) dissociated cells and (B) aggregated cell) and scanning electron microscopy images ((C) basement membrane-like matrix, (D) aggregated cells, and (E) Transwell® insert membrane) of hiPSC-RPE (454E2) treated with 2000 *μ*g/ml rtPA. Scale bar, 10 mm.

To elucidate the consistency of response to rtPA between hfRPE and hiPSC-RPE, we evaluated the effects of 24-hour rtPA exposure on cell morphology and RPE-specific cell functions. The hfRPE and both hiPSC-RPE were cultured with four different dilutions of rtPA (0, 20, 100, and 2000 *μ*g/ml) for 24 hours. All RPE treated with 20 (clinically used concentrations) and 100 (clinically toxic concentrations) *μ*g/ml rtPA expressed the tight junction marker ZO-1 in an immunocytochemistry assay (green: ZO-1, blue: nuclei) and showed no observable morphological changes under scanning electron microscopy (Figures 3(a)–3(c)). In 2000 *μ*g/ml, rtPA-treated hfRPE and both hiPSC-RPE, dissociated cells, and no expression of ZO-1 were detected, and scanning electron microscopy revealed basement membrane-like matrix with aggregated cells (Figure 3(d)). Based on these findings, rtPA at 20 and 100 *μ*g/ml does not obviously affect RPE cell morphology.

**Figure 4:**
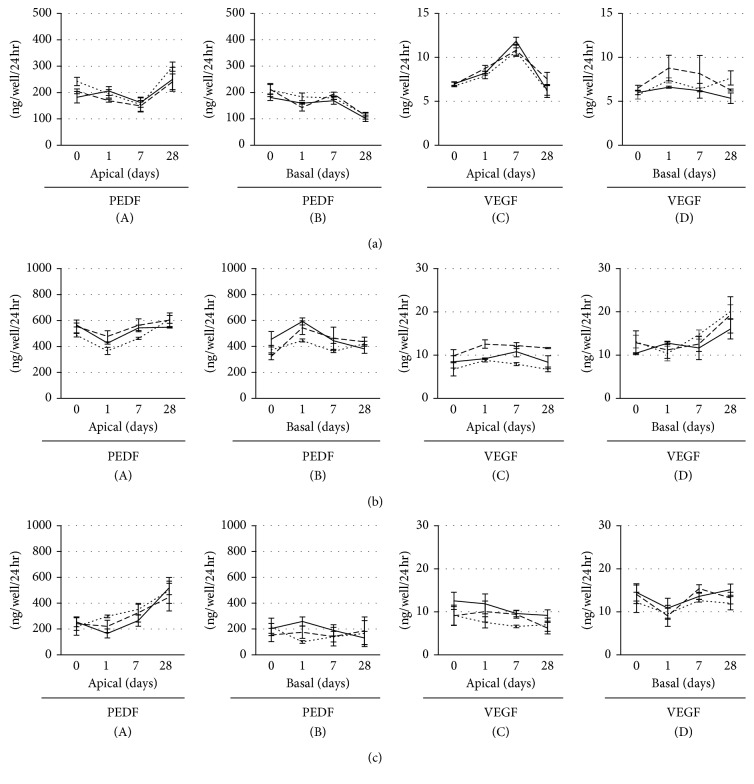
Effects of 24-hour rtPA exposure on PEDF and VEGF secretion. The secretion of PEDF ((A) apical side and (B) basal side) and VEGF ((C) apical side and (D) basal side) in hfRPE (a), hiPSC-RPE ((b) 454E2), and hiPSC-RPE ((c) 253G1) treated with three different dilutions (solid line: 0 *μ*g/ml, dotted line: 20 *μ*g/ml, and dashed line: 100 *μ*g/ml); *n*=3 for each; ^*∗*^*P* < 0.01.

RPE secrete a range of growth factors, such as PEDF and VEGF, that maintain retinal and choroidal homeostasis. Therefore, we investigated whether 20 and 100 *μ*g/ml rtPA affected PEDF and VEGF secretion. HfRPE and both hiPSC-RPE were cultured on Transwell® inserts in the medium containing three different dilutions of rtPA (0, 20, and 100 *μ*g/ml). PEDF and VEGF concentrations in the apical and basal media of RPE cells grown in these inserts were measured by ELISA at 1, 7, and 28 days after tPA administration, and no significant differences were observed at all time points (Figure 4(a): hfRPE, Figure 4(b): hiPSC-RPE (454E2), and Figure 4(c): hiPSC-RPE (253G1)).

**Figure 5:**
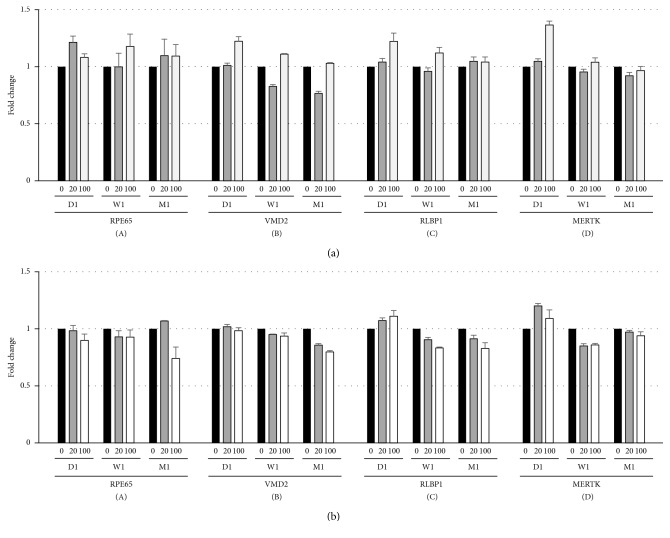
Effects of 24-hour rtPA exposure on RPE-specific gene expression. Time course (D1: day 1; W1: day 7; M1: day 28) of relative RPE-specific gene expression of hiPSC-RPE ((a) 454E2 and (b) 253G1) treated with three different dilutions (black bar: 0 *μ*g/ml; brown bar: 20 *μ*g/ml; white bar: 100 *μ*g/ml); *n*=3 for each; ^*∗*^*P* < 0.01.

Next, we investigated whether rtPA affects RPE-specific gene expression (RPE65 [17], VMD2 [18], RLBP1 [19], and MERTK [20]). HfRPE and both hiPSC-RPE were cultured in the medium containing three different dilutions of rtPA (0, 20, and 100 *μ*g/ml), and RPE-specific gene expression was evaluated by performing real-time qRT-PCR at 1, 7, and 28 days after rtPA administration. Expression levels of all RPE hallmark genes in 20 and 100 *μ*g/ml rtPA-treated hiPSC-RPE were comparable to those in control (Figure 5(a): hiPSC-RPE (454E2) and Figure 5(b): hiPSC-RPE (253G1)) and increased in a time-dependent manner (data not shown). We obtained similar results with hfRPE (data not shown). Based on these results, 20 and 100 *μ*g/ml rtPA do not inhibit typical RPE function.

## 4. Discussion

The damaging effects of subretinal hemorrhage on the retina are attributed to the release of toxic substances such as fibrin [21], iron [22], and haemosiderin [23], limited nutrient and metabolite diffusion, and traction of the neural retina [24]. Traditionally, subretinal hemorrhage was directly removed [25]; however, this method requires an invasive procedure, such as a large retinotomy and the inadvertent removal of corresponding RPE. To overcome these disadvantages, new methods to address subretinal hemorrhage was introduced, such as intravitreal injection of rtPA and gas [7] or vitrectomy, followed by subretinal rtPA injection and gas tamponade [6] to displace the hemorrhage from the submacular region. Although rtPA-assisted subretinal hemorrhage displacement leads to improved visual prognosis, additional retinal complications resulting from rtPA cytotoxicity were reported clinically in [7, 9]. To our knowledge, there are no published reports investigating the RPE toxicity of rtPA in vitro. The human RPE cell line ARPE19 has been used for preclinical pharmaceutical evaluation. Since ARPE19 expresses RPE-specific markers and may be grown in culture for prolonged periods, it is a critical tool for RPE cell biology. However, immortalization cells ARPE19 potentially show different experimental responses compared to native RPE. Therefore, another cell source to improve cytotoxicity testing accuracy is required. The present study reported the responsiveness of hiPSC-RPE, hfRPE, and ARPE19 to rtPA in terms of cell morphology, cell death, and cell function to conceptually validate drug-induced cytotoxicity testing using hiPSC-RPE. The rtPA-induced cell damage in both hiPSC-RPE was similar to that observed in hfRPE, while the responses of ARPE19 significantly differed from hfRPE. Previously, we classified 12 hiPSC-RPE, 3 hfRPE, ARPE19, and 12 fibroblast cell lines using microarray data generated with 54,675 probe sets and constructed phylogenetic trees [3]. This analysis revealed that all hiPSC-RPE grouped near the hfRPE cluster, whereas ARPE19 was located near the fibroblast cluster. In addition, we examined the expression of 154 RPE signature genes [26] in 12 hiPSC-RPE, 3 hfRPE, and ARPE19 cell lines. All hiPSC-RPE exhibited similar expression patterns to hfRPE, whereas many genes in ARPE19, including critical genes such as RPE65 and BEST1, had lower expression than hfRPE. Thus, hiPSC-RPE are a cell source for in vitro cytotoxicity testing to circumvent the inaccuracies associated with ARPE19.

rtPA converts plasminogen to plasmin, resulting in clot lysis; thus, rtPA needs to touch subretinal hemorrhage directly. The neural retina contains membrane tissues formed by Müller cells, termed the inner limiting membrane (ILM) and external limiting membrane (ELM), which restrict the diffusion of external substances in the retina. According to previous reports, ILM prevents the diffusion of rtPA (70 kDa) but not 20 kDa dextran [27], whereas ELM blocks proteins greater than 36 Å (60 kDa) in normal rabbit retina [28]. The 70 kDa molecular weight of rtPA theoretically renders it unable to diffuse into the subretinal space from the vitreous cavity; however, the intravitreal injection of rtPA passed through rabbit retina afflicted by subretinal hemorrhage and dissolved subretinal hemorrhage in clinical settings. Therefore, drug diffusion and excretion in vivo are strongly affected by tissue conditions such as disease severity and temporal changes, making it difficult to establish fixed in vitro drug conditions, such as concentration or exposure time. Based on previous clinical reports, rtPA at a dose greater than 100 *μ*g/ml appears to be toxic to the neural retina and RPE. Hesse et al. [7] demonstrated exudative retinal detachment followed by hyperpigmentation of the RPE in all eyes treated with 100 *μ*g of rtPA (4/4) but not in eyes that received 50 *μ*g of rtPA (0/7). Additionally, Chen et al. [9] reported a case of diffuse pigmentary alterations, sparing the posterior pole after two intravitreal injections of 50 *μ*g of rtPA. In this study, rtPA exhibited time-dependent RPE toxicity; hiPSC-RPE and hfRPE treated with 50 and 100 *μ*g/ml rtPA showed no cytotoxicity after rtPA exposure for 1 hour, whereas 24-hour rtPA exposure caused RPE toxicity at the clinical concentration of 20 *μ*g/ml as well as 50 and 100 *μ*g/ml rtPA. These results suggested that clinical rtPA-induced RPE toxicity occurs between 1 hr and 24 hr rtPA (100 *μ*g/ml) exposures in vitro. The exposure of 100 *μ*g/ml rtPA for 24 hours induced no cytotoxicity in terms of RPE cell morphology, VEGF and PEDF secretion, and RPE-specific gene expression. Thus, 20 *μ*g/ml rtPA used in clinical is the safe concentration, and it is reasonable to assess drug-induced cytotoxicity using in vitro concentrations estimated by comparing in vitro and in vivo results.

One limitation of our study is the use of human fetal RPE as control, which may influence the outcomes due to cell immaturity. We showed that the responsiveness of hiPSC-RPE to rtPA, but not ARPE19 (adult RPE), were similar to that of hfRPE, suggesting that the RPE in patients could show different responsiveness. However, in the previous report [26], the gene-expression of fetal native RPE, adult native RPE, fetal cultured RPE, and adult cultured RPE (ARPE19) were evaluated, and native cells (fetal and adult native RPE) and cultured cells (fetal cultured RPE and ARPE19) clustered separately regardless of cell source (fetal or adult). Moreover, the cadaveric eyes (adult RPE) are exposed to long-term hypoxic stress, which could change the state of cells. The cytotoxicity testing in vitro is performed using cultured cells, and thus, we used hfRPE as a control. The use of fixed culture condition, which was optimized for the differentiation into RPE from hiPSCs, is another potential limitation. The cell characteristics alter depending on culture conditions; thus, different culture conditions could induce similar response between hfRPE and ARPE19 in cytotoxicity testing. In addition, the RPE of patients with subretinal hemorrhage is affected by blood components or external pressure. These conditions might adversely affect the cell function as well as cell death, and patient RPE may be even more affected by rtPA.

## 5. Conclusions

We report here that the responsiveness of hiPSC-RPE to rtPA is remarkably similar to that of hfRPE in terms of cell morphology, cell death, and cell function. These findings suggest that hiPSC-RPE is a valuable cell source for further in vitro cytotoxicity testing. In addition, the concentration of rtPA estimated by comparing in vitro and in vivo results induced no cytotoxicity in terms of RPE cell morphology, VEGF and PEDF secretion, and RPE-specific gene expression. The clinically used rtPA-assisted displacement of subretinal hemorrhage is a safe treatment method. These results provide additional information supporting the use of hiPSCs for drug development.

## Data Availability

Data are available from Hiroyuki, Kamao MD, PhD (hironeri@med.kawasaki-m.ac.jp), for researchers who meet the criteria for access to confidential data.
